# Enzyme Degradation Reagents Effectively Remove Mycotoxins Deoxynivalenol and Zearalenone from Pig and Poultry Artificial Digestive Juices

**DOI:** 10.3390/toxins11100599

**Published:** 2019-10-15

**Authors:** Ko-Hua Tso, Jyh-Cherng Ju, Yang-Kwang Fan, Hsin-I Chiang

**Affiliations:** 1 Department of Animal Science, National Chung Hing University, Taichung 40227, Taiwan; d100037004@mail.nchu.edu.tw (K.-H.T.); jcju@dragon.nchu.edu.tw (J.-C.J.); ykfan7712@gmail.com (Y.-K.F.); 2 Graduate Institute of Biomedical Sciences, China Medical University, Taichung 40402, Taiwan; 3 Translational Medicine Research Center, China Medical University Hospital, Taichung 40402, Taiwan; 4 Department of Bioinformatics and Medical Engineering, Asia University, Taichung 41354, Taiwan

**Keywords:** adsorbent, deoxynivalenol, enzyme degradation reagent, in vitro, zearalenone

## Abstract

Mycotoxin removers include enzymes and adsorbents that may be used in animal feeds to eliminate the toxic effects of mycotoxins. This study aimed to determine the removability of two different types of mycotoxin removers, adsorbents and enzyme degradation reagents (EDRs), in the simulated gastrointestinal conditions of pigs and poultry. Seven commercial mycotoxin removers, including five EDRs and two adsorbents, were tested in vitro. In this study, the supplemented dosages of mycotoxin removers used in pig and poultry feeds were the commercial recommendation ranging from 0.05% to 0.2%. For pigs, the in vitro gastric and small intestinal simulations were performed by immersing the mycotoxin-tainted feed in artificial gastric juice (AGJ) at pH 2.5 for 5 h or in artificial intestinal juice (AIJ) at pH 6.5 for 2 h to mimick in vivo conditions. For poultry, mycotoxin-tainted feeds were immersed in AGJ for 2 h at pH 4.5 and 0.5 h at pH of 2.5, respectively, to simulate crop/glandular stomach and gizzard conditions; the small intestinal simulation was in AIJ for 2 h at pH 6.5. For the pig, EDRs and adsorbents had deoxynivalenol (DON) removability (1 mg/kg) of 56% to 100% and 15% to 19%, respectively. Under the concentration of 0.5 mg/kg, the zearalenone (ZEN) removability by EDRs and adsorbents was 65% to 100% and 0% to 36%, respectively. For the simulation in poultry, the removability of DON by EDRs and adsorbents (5 mg/kg) was 56% to 79% and 1% to 36%, respectively; for the concentration of 0.5 mg/kg, the removability of ZEN by EDRs and adsorbents was 38% to 69% and 7% to 9%, respectively. These results suggest that EDRs are more effective in reducing DON and ZEN contamination compared to the adsorbent methods in the simulated gastrointestinal tracts of pig and poultry. The recoveries of DON and ZEN of pig in vitro gastrointestinal simulations were higher than 86.4% and 84.7%, respectively, with 88.8% and 85.9%, respectively, in poultry. These results demonstrated the stability and accuracy of our mycotoxin extraction process and in vitro simulation efficiency.

## 1. Introduction

Mycotoxins are toxic and complicated secondary active biological metabolites produced by filamentous fungal species, mainly *Aspergillus*, *Fusarium,* and *Penicillium* [1]. When animals consume feeds contaminated with mycotoxins, they suffer from a series of toxic effects, such as decreased feed intake, reduced body weight gain, diarrhea, vomiting, as well as liver and kidney pulmonary edema [2]. Many different strategies, such as thermal inactivation, irradiation, physical dilution, and mycotoxin removers (adsorbents or degradation reagents), are used in feed mills and farms to reduce mycotoxin concentration of feedstuffs and feeds [3]. However, mycotoxins are heat resistant, and this property makes it difficult to completely eliminate them during the food production process [4,5]. Ultraviolet (UV) irradiation [3] and moldy grain diluted with a quantity of clean grain [6] are the most frequently used non-thermal feed and food processing methods. These methods also have many limitations, such as low efficacy, lengthy treatment duration, and the expense of equipment required to implement these techniques. Feed-processing machines and storage silos normally operate at room temperature with no ventilation. These conditions often lead to mold growth and mycotoxin contamination, resulting in great concerns for feed and animal safety [7].

Adding mycotoxin removers in feeds is currently an effective strategy for detoxifying animal feeds that contain moderate to low levels of mycotoxins [8]. There are two major types of mycotoxin removers, i.e., adsorbents and enzyme degradation reagents (EDRs). Mycotoxin adsorbents work by preventing the absorption of mycotoxins by the gastrointestinal tract of the animal by adsorbing the toxins to their surfaces. They are either inorganic (e.g., bentonites and hydrated sodium calcium aluminosilicate) or organic products (yeast cell walls) [9,10]. Enzyme degradation reagents used in this study are defined as the biodegradation of the toxic chemical structure of the mycotoxins into non-toxic metabolites by using microorganisms and their metabolites or specifically extracted components, such as enzymes [11].

Mycotoxins are classified into polar and non-polar categories according to their chemical structures [12]. Polar mycotoxins, such as aflatoxin B_1_ (AFB_1_) and fumonisin B1 (FB_1_), are more easily adsorbed by adsorbents than non-polar mycotoxins. On the other hand, the solubility, molecular weight of mycotoxins—in the case of ionized compounds—charge distribution and dissociation constants play a critical role in mycotoxin adsorbent ability [13]. AFB_1_ has a higher adsorption rate than FB_1_ by adsorbents. Therefore, AFB_1_ is commonly used as a target for evaluating the mycotoxin removability of adsorbents [14]. Non-polar mycotoxins include ochatoxin A, T-2 toxin, deoxynivalenol (DON) and zearalenone (ZEN) [15,16]. Monogastric animals, such as pigs and poultry, are particularly vulnerable to mycotoxins because of the high proportion of cereals in their diets and the lack of rumen with microbiota capable of degrading mycotoxins before intestinal absorption [17]. Zearalenol and its metabolites are generally hypothesized to mimic estrogen-like actions and compete with estrogen in binding to estrogen receptors in gilts and sows [18]. Sows and gilts have a lower tolerance of ZEN toxicosis than laying poultry. Typical symptoms of ZEN toxicosis in gilts and sows are reddening, hyperemia, and edematous swelling of the vulva, enlargement of the uterus with the formation of cysts on the ovaries and enlargement of the mammary glands [19]. Both pigs and poultry are very susceptible to DON. DON inhibited protein synthesis [20], and the main symptoms of DON toxicosis are growth performance reduction, diarrhea, and gastrointestinal necrosis in animals [1]. Most mycotoxin adsorbents have demonstrated lower performance (or even lack of performance) in terms of the ability to adsorb DON and ZEN [11]. On the other hand, biological degradation of DON and ZEN by EDRs is likely a suitable way to control their toxicities as supported in many previous studies [21,22]. At present, most of the commercial EDRs have both adsorbents and enzymes, because they have different functions for removing polar mycotoxin (e.g., AFB_1_) and non-polar mycotoxin (e.g., DON and ZEN).

Removability is commonly used as a standard measurement for assessing the removal efficiency of mycotoxins, but to date, a robust method for the evaluation of mycotoxin removers is currently unavailable. Traditionally, the mycotoxin removability was determined by using both in vitro and in vivo assays. In vitro and in vivo experiments have both advantages and disadvantages. For in vivo studies, individual bioassays should be conducted using the same strain, age, body weight, and diet of animals to obtain consistent results. Variations in housing conditions, health status, growth rate, and maturity of animals also exert influences. In contrast, in vitro experiments are relatively easy to perform, and they can greatly shorten the time and cost of experimentation. Most feed mills still use in vitro methods to screen suitable mycotoxin removers due to the concerns regarding the cost in time, animals, manpower and feeds. However, the ultimate goal of an in vitro study is to replace in vivo experiments in general. Therefore, the conditions of in vitro experiments should be tightly controlled and well-designed to closely resemble the natural environments of the target animal species, in turn, leading to the high reproducibility of research data [23].

In general, the in vitro method uses a simple buffer (such as phosphate buffer solution) instead of digestive juice as the buffered matrix. It was widely used in evaluating the mycotoxin removal ability of adsorbents, although it has lower reliability and consistency compared to in vivo tests [24]. Similar models have been successfully applied to previous in vitro simulations of mycotoxin removers [25,26]. However, these models mainly differed in certain aspects, including simulation media or solutions (pH value and composition) and types of mycotoxin removers (EDRs or adsorbents). In addition, temperature, endogenous digestive enzymes, and agitation speed to simulate gastrointestinal motility are also important factors that delineate the in vitro from the in vivo efficacy.

Ruminants are generally considered to be less susceptible to the harmful effects of mycotoxins than monogastric animals [17]. The assessment of mycotoxin removability is mostly limited to the gastrointestinal tracts in monogastric animals [27]. The gastrointestinal tract conditions of pigs and poultry differ considerably. According to the reports from Zyla et al. [25] and Avantaggiato et al. [28], in vitro simulation conditions in monogastric animals are usually set at pH 2.3 to 2.5 and maintained for 3 to 5 h to match pig stomach conditions; to match pig intestinal conditions, conditions are set at pH 6.2 to 6.5 and maintained for 6 h. For poultry, the conditions are set at pH 4.5, maintained for 2 h for poultry crop and glandular stomach simulation; pH 2.5, maintained for 0.5 h for poultry gizzard simulation; and pH 6.2 to 6.5 maintained for 3 h for poultry intestinal simulation. Most mycotoxins are absorbed in the upper part of the small intestines [29]. The simulation of in vitro conditions is focused on the environments of the stomach and small intestine. In addition, the pH value, time, temperature, animal endogenous digestive enzymes, and agitation speed (simulating gastrointestinal motility) are among the critical factors that influence the difference between in vitro and in vivo conditions.

The dosages of mycotoxin removers used in this study were determined according to the recommended dosages by manufacturers (0.05% to 0.2% of each mycotoxin remover, Table 1). In general, the overall mycotoxin remover levels did not exceed 0.2%, which was recognized as safe (GRAS) for animal feeds by the United States Food and Drug Administration (FDA) [30]. In this study, we used ZEN concentrations of 0.5 mg/kg for both pig and poultry feeds, where different DON concentrations were used for pig (1 mg/kg) and poultry (5 mg/kg) feed. The dosages used were designed based on the report of China Hygienic Standard for Feed (GB13078-2017) and FDA regulations [31].

Commonly mycotoxin analysis methods include enzyme-linked immunosorbent assays (ELISA), high-performance liquid chromatography (HPLC), and LC-MS/MS. The ELISA have been routinely used for quick evaluation of mycotoxins. The method has shown with reliable accuracy only in simple substrates, i.e., feed ingredients, but not in complex substrates, e.g., formulated feeds suspended in the digestive juice [26]. High-performance liquid chromatography and LC-MS/MS-based analyses are relatively more accurate than rapid test kits and ELISA for evaluating mycotoxin removers. The limit of detection (LOD) and accuracy of LC-MS/MS is better than HPLC [26]. However, HPLC is more commonly used as a reference method for in vitro analysis of mycotoxin removal reagents [32,33]. High-performance liquid chromatography is used as an analytical standard for detecting mycotoxin in many countries (prEN 15791:2009; prEN 15792:2009). The technical requirements of the operator and high equipment cost of LC-MS/MS makes this method less accessible than HPLC in feed mills or the regular analytical laboratories. The LOD of HPLC fits the requirements of DON and ZEN analysis of in vitro simulation (prEN 15791:2009; prEN 15792:2009). For the above reason, HPLC, instead of LC-MS/MS as a preferred methodology for in vitro analysis of mycotoxin, is considered as the most appropriate, accurate, and reliable procedure to monitor the efficacy of mycotoxin remover reagent at industrial levels, as well as for law enforcement. In terms of accuracy and precision, we also determined the recoveries of DON and ZEN, which standards were added to the feed. The contaminated feeds were examined in gastrointestinal in vitro simulations. The recovery rate was calculated by comparing the peak areas of mycotoxin standards and mycotoxins in the feed released in the simulated digestive juice.

To improve the accuracy of assessing mycotoxin removability, we referred to many reports and modified the in vitro simulation conditions to be closer to the pig gastrointestinal tract in nature [26,28]. Furthermore, we also modified the simulation conditions of poultry gastrointestinal tracts based on the report by Zyla et al. [25]. Pig and poultry gastrointestinal environments were simulated using artificial gastric juices (AGJ) and artificial intestinal juices (AIJ), and mycotoxin removability of the two types of mycotoxin removers (adsorbents and EDRs) on DON- or ZEN-inoculated commercial feeds was evaluated using HPLC.

## 2. Results

### 2.1. Simulation of Pig Gastrointestinal Tracts

**Figure 1:**
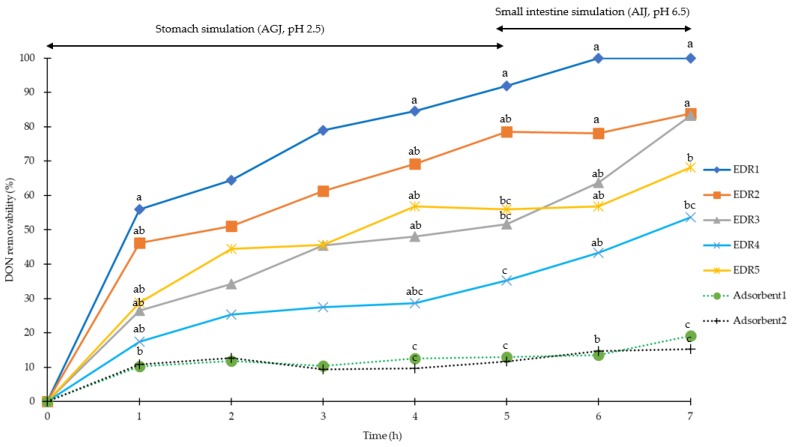
Removability of deoxynivalenol (DON) (1 mg/kg) with enzyme degradation reagents (EDRs) (solid line) and adsorbents (dotted line) in pig gastrointestinal simulations (0 to 5 h for stomach simulation, 6 to 7 h for small intestine simulation). ◆:0.05% EDR1; ■: 0.1% EDR2; ▲: 0.1% EDR3; ×: 0.1% EDR4; □: 0.2% EDR5; ●: 0.2% Adsorbent1; ＋: 0.2% Adsorbent2. ^a,b,c^ Means of the same column (time point) without the same superscripts differ (*p* < 0.05). AGJ: Artificial gastric juice; AIJ: Artificial intestinal juice; DON: Deoxynivalenol; EDR: Enzyme degradation reagents; ZEN: Zearalenone.

In terms of accuracy (denoted as the recovery rate) and precision (denoted as the deviation of replicates), the DON recoveries from AGJ and AIJ were over 90.0% and 86.4%, respectively (with coefficient of variation (CV) < 5% for AGJ and AIJ), as shown in Table 2. For the simulated pig stomach conditions (pH of 2.5) for 5 h, the removabilities of DON at the 1 mg/kg level were 92%, 79%, 52%, 35%, and 56% for EDRs (1 to 5), respectively, and 12% and 13% for Adsorbent1 and Adsorbent2, respectively. For the simulated pig small intestine conditions (pH of 6.5) for 2h, the removabilities of DON at 1 mg/kg level were 100%, 84%, 83%, 54%, and 68% for EDRs (1 to 5), respectively, and 15% and 19% for Adsorbent1 and Adsorbent2, respectively (Figure 1). After 5 h of stomach simulation, all the EDRs, except EDR4, showed higher DON removability than both of the adsorbents (*p* < 0.05), and the removability of EDR1 was higher than all other EDRs (*p* < 0.05) except for EDR2. No significant difference in removability was observed between the two adsorbents. After 2 h of small intestine simulation, all EDRs, except for EDR4, had higher DON removability than both adsorbents (*p* < 0.05), and EDR1 was higher than EDR5 and EDR6 (*p* < 0.05), with no significant difference of removability being found between two adsorbents.

**Figure 2:**
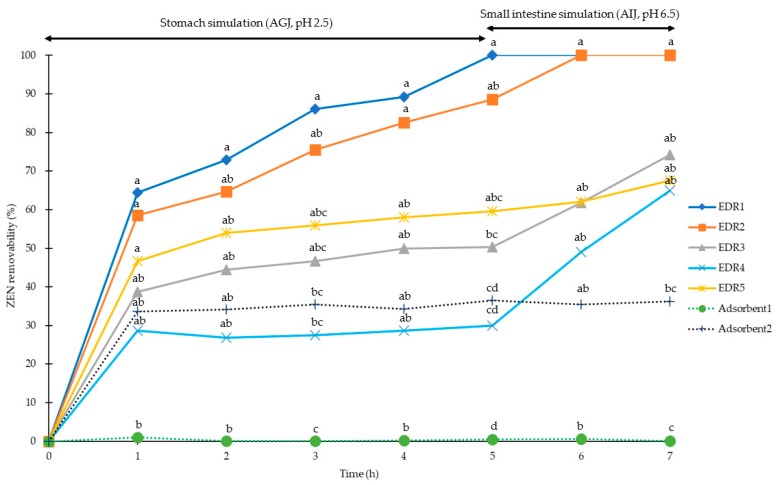
Removability of zearalenone (ZEN) (0.5 mg/kg) with EDRs (solid line) and adsorbents (dotted line) in pig gastrointestinal simulations (0 to 5 h for stomach simulation, 6 to 7 h for small intestine simulation). ◆:0.05% EDR1; ■: 0.1% EDR2; ▲: 0.1% EDR3; ×: 0.1% EDR4; □: 0.2% EDR5; ●: 0.2% Adsorbent1; ＋: 0.2% Adsorbent2. ^a,b,c,d^ Means of the same column (time point) without the same superscripts differ (*p* < 0.05). AGJ: Artificial gastric juice; AIJ: Artificial intestinal juice; DON: Deoxynivalenol; EDR: Enzyme degradation reagents; ZEN: Zearalenone.

The ZEN recoveries of AGJ and AIJ were higher than 92.5% and 84.7%, respectively (with CV < 5 for both AGJ and AIJ), as shown in Table 2. For the stimulated pig stomach conditions (pH of 2.5), the removabilities of ZEN at 0.5 mg/kg level were 100%, 89%, 60%, 50%, and 37% for EDRs (1 to 5), and 0% and 30% for Adsorbent1 and Adsorbent2, respectively. For the stimulation of pig small intestine conditions (pH of 6.5), the removabilities of ZEN at 0.5 mg/kg were 100%, 100%, 74%, 68%, and 65% for EDRs (1 to 5), respectively, and 0% and 36% for Adsorbent1 and Adsorbent2, respectively (Figure 2). After 5 h of stomach simulation, only EDR1 and EDR2 had higher ZEN removal ability than the two adsorbents (*p* < 0.05), and ZEN removability of EDR1 was higher than EDR3 and EDR4 (*p* < 0.05). After 2 h of small intestine simulation, only EDR1 showed higher ZEN removability than in both adsorbents (*p* < 0.05).

### 2.2. Simulation of Poultry Gastrointestinal Tracts

**Figure 3:**
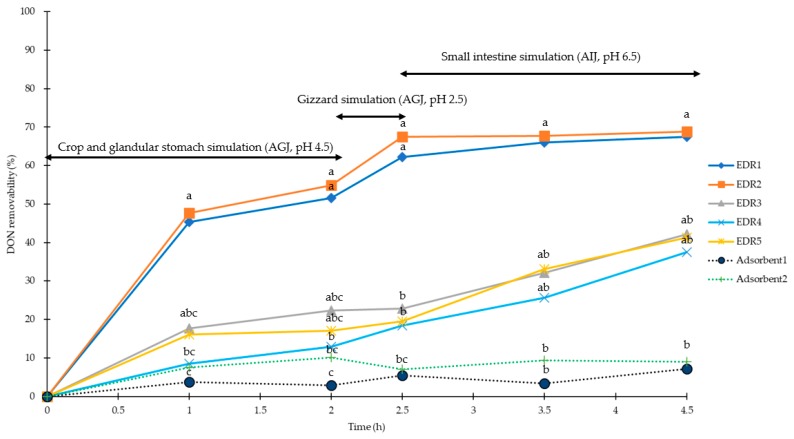
Removability of DON (5 mg/kg) with EDRs (solid line) and adsorbents (dotted line) in chicken gastrointestinal simulations (0 to 2 h for crop and glandular stomach simulation, 2 to 2.5 h for gizzard simulation, 2.5 to 4.5 h for small intestine simulation). ◆:0.05% EDR1; ■: 0.1% EDR2; ▲: 0.1% EDR3; ×: 0.1% EDR4; □: 0.2% EDR5; ●: 0.2% Adsorbent1; ＋: 0.2% Adsorbent2. ^a,b,c^ Means of the same column without the same superscripts differ (*p* < 0.05). AGJ: Artificial gastric juice; AIJ: Artificial intestinal juice; DON: Deoxynivalenol; EDR: Enzyme degradation reagents; ZEN: Zearalenone.

In the present study, the efficiencies of DON recovered from AGJ (pH 4.5 and 2.5) and AIJ (pH 6.5) were higher than 91.9%, 89.8%, and 88.8%, respectively, as shown in Table 2 (CV < 7% for AGJ and AIJ). For the stimulated poultry crop and glandular stomach conditions (pH of 4.5) for 2 h, the removabilities of DON (5 mg/kg) by EDRs (1 to 5) were 52%, 55%, 22%, 13%, and 17%, respectively, and 3% and 10% for Adsorbent1 and Adsorbent2, respectively. For the simulated poultry gizzard conditions (pH of 2.5) for 0.5 h, the removabilities of DON at 5 mg/kg level were 62%, 67%, 23%, 18%, and 20% by EDRs (1 to 5), respectively, and 5% and 7% for Adsorben1 to Adsorbent2, respectively. For the simulated poultry small intestine conditions (pH of 6.5) for 2 h, the removabilities of DON at 5 mg/kg were 68%, 69%, 42%, 41%, and 38% for EDR1 to EDR5, respectively, and 7% and 9% for Adsorbent1 to Adsrobent2, respectively as shown in Figure 3. At the 2 h simulation in crop and glandular stomach, only EDR1 and EDR2 showed significantly higher DON removabilities than both adsorbents (*p* < 0.05), and the DON removability of EDR4 was lower than that of EDR1 and EDR2 (*p* < 0.05). No significant difference was observed between the two adsorbents. When gizzard simulation was performed for 0.5 h, EDR1 and EDR2 showed higher removability for DON than that in all other EDRs and adsorbents (*p* < 0.05), and EDR1 had significantly higher removability than EDR3, EDR4 and EDR5 (*p* < 0.05); in addition, no difference was found between other EDRs and the two adsorbents. Two hours after the small intestine simulation, only EDR1 and EDR2 had higher DON removability than that of the adsorbents (*p* < 0.05). There was no difference between other EDRs and adsorbents, either.

**Figure 4:**
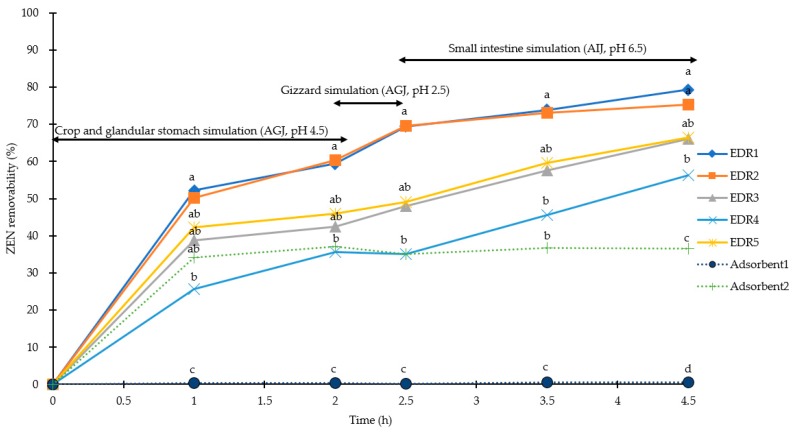
Removability of ZEN (0.5 mg/kg) with EDRs (solid line) and adsorbents (dotted line) in poultry gastrointestinal simulations (0 to 2 h for crop and glandular stomach simulation, 2 to 2.5 h for gizzard simulation, 2.5 to 4.5 h for small intestine simulation). ◆:0.05% EDR1; ■: 0.1% EDR2; ▲: 0.1% EDR3; ×: 0.1% EDR4; □: 0.2% EDR5; ●: 0.2% Adsorbent1; ＋: 0.2% Adsorbent2. ^a,b,c,d^ Means of the same column (time point) without the same superscripts differ (*p* < 0.05). AGJ: Artificial gastric juice; AIJ: Artificial intestinal juice; DON: Deoxynivalenol; EDR: Enzyme degradation reagents; ZEN: Zearalenone.

The efficiencies of ZEN recovered from AGJ at pH 4.5 and 2.5 and AIJ at pH 6.5 of were higher than 90.0%, 87.3%, and 85.9% (CV < 7 for AGJ and AIJ), respectively (Table 2). For the stimulated poultry crop and glandular stomach conditions (pH of 4.5), the removabilities of ZEN at 0.5 mg/kg were 59%, 60%, 43%, 36%, and 46% by EDRs (1 to 5), respectively, and 0% and 37% by Adsorbent1 and Adsorbent2, respectively. Under the simulated poultry gizzard conditions (pH of 2.5), the removabilities of ZEN at 0.5 mg/kg were 69%, 70%, 48%, 35%, and 49% by EDRs (1 to 5), respectively, and 0% and 35% for Adsorben1 and Adsorbent2, respectively. For the simulated poultry small intestine conditions (pH of 6.5), the removabilities of ZEN (0.5 mg/kg) were 79%, 75%, 66%, 56%, and 66% by EDRs (1 to 5), respectively, and 1% and 36% by Adsorbent1 and Adsrobent2, respectively (Figure 4). During 2 h of simulation in the crop and the glandular stomach, only EDR1 and EDR2 had higher efficiencies of ZEN removability than both Adsorbents (*p* < 0.05), but no difference was observed between other EDRs and Adsorbent2. The ZEN removability of Adsorbent2 was higher than that of Adsorbent1 (*p* < 0.05). During pH value diversification of three artificial digestive juices, EDR1 and EDR2 maintained the highest ZEN removability than other EDRs and adsorbents, with EDR4 being the lowest. All EDRs showed significantly higher ZEN removability than the two adsorbents (*p* < 0.05).

## 3. Discussion

In vitro simulation methods need to be matched with appropriate mycotoxin quantification protocols to obtain accurate results [26]. In our study, we used external standards for DON and ZEN, and the recoveries of DON and ZEN were higher than 80% with the CVs less than 10% in both AGJ and AIJ of pig and poultry gastrointestinal simulations. Recoveries and CVs of our results were higher than those published by Wang et al. [26] using external standards. In addition, the CV of our measurements was lower, probably due to the immunoaffinity columns applied in the present study, so the noise interference was reduced from the buffered matrix. The treatment of immunoaffinity columns may play a more critical role when an LC-MS/MS technique is used, as it has a higher sensitivity than the HPLC technique for mycotoxin quantification [34].

Our results indicated that DON and ZEN removabilities of two adsorbents were both lower than 40% in the pig and the poultry simulation models, in agreement with the results of Wang et al. [26]. It is shown that the mineral binders had binding efficacies of 8.11% to 14.71%, and 13.67% to 29.97% for DON and ZEN in AGJ, as well as 12.29% to 31.31%, and 0% to 23.16% in AIJ, respectively. To date, there is no in vitro report for detecting DON and ZEN removabilities of EDRs, only in vivo reports are available [35,36]. It is because previous in vitro simulations methods only used simple buffer solutions [33,37], which may not be a suitable condition for the enzymatic reactions. According to the patents of EDR5 (Table 1), the DON and ZEN removabilities of EDR5 in artificial digestive juice for 72 h were 95% and 85%, respectively. In our pig (7 h) and poultry (4.5 h) digestive simulation, the DON and ZEN removabilities of EDR5 were slightly less than 60%, which was close to the results of the patented efficacy taking into account the shorter experimental times.

DON and ZEN removabilities for the two adsorbents in AGJ and in AIJ were both lower than 40% in the present study. No significant effect of pH on the removabilities of DON and ZEN by the adsorbents was observed, but their removability significantly increased over time. Similar results have been reported in previous studies [24,26,37]. De Mill et al. [37] claimed that pH level may influence the phenolic hydroxyl group of ZEN and DON, or the ionization-state of the functional groups of adsorbents and alter the chemical sorption due to the ionic interaction. Low pH can also facilitate degradation of the minerals, and this effect is mostly seen in longer treatment periods. However, in our study, the DON and ZEN removabilities did not significantly differ between long and short treatment periods (2.5 to 5 h). Furthermore, polar mycotoxins (such as AFB_1_) are more hydrophilic than non-polar mycotoxins (such as ZEN and DON). Since the interlayer spaces of most adsorbents are hydrophilic [37], adsorbents interact with polar mycotoxin more actively than with non-polar mycotoxins. The commercial EDRs used in this research included adsorbents and mycotoxin degradation enzymes (Table 1). In general, high adsorption rates have been reported in vitro as well as in vivo for polar mycotoxin (such as AFB_1_) [38] by adsorbents. However, the main disadvantage of adsorbents is nutritional losses, such as vitamin B_6_, manganese, and zinc [39]. Some studies have also indicated that the application of enzymes is an alternative method for detoxifying AFB_1_ in food and feed, such as laccase and manganese peroxidase [40,41]. The compositions of the commercial EDRs used in this research did not contain AFB_1_-degraded enzymes, except EDR5. Zhang et al. [42] indicated *Bacillus subtilis ANSB060* of EDR5 could degrade AFB_1_ and reduce the toxicities of AFB_1_ in duck, but the composition of EDR5 still have an adsorbent (yeast cell wall). Above all, the commercial EDRs used in this research rely on adsorbents to not bind AFB_1_ on mycotoxin degradation enzymes.

In the current study, we found that even with the dosage of adsorbents being two to four folds higher than EDRs, their removabilities for DON and ZEN were still lower than that of EDRs. This result is probably because zeolites and hydrated sodium calcium aluminosilicate (HSCAS) are probably not structurally fit for DON and ZEN adsorption. Many in vitro and in vivo studies indicated that it is more difficult to remove non-polar mycotoxins, such as DON and ZEN [11,13,38], by adsorbents. The results of Wang et al. [26] indicated the DON and ZEN removabilities of mineral binders were lower than 15% and 30% in AGJ, as well as lower than 32% and 24% in AIJ, respectively. Similarly, the DON and ZEN removabilities of yeast cell wall were lower than 17% and 39% for DON in AGJ, as well as lower than 24% and 35% in AIJ, respectively [26]. Therefore, although the commercial EDRs examined in this study also contain adsorbents, the proportion of adsorbents in the commercial EDRs is lower than 20%. Based on the reasons above, we hypothesized that the adsorbents of these commercial EDRs have an extremely minimal influence on DON and ZEN removabilities.

Effective mycotoxin degradation enzymes transform the toxic structure of target mycotoxins. Many studies have indicated the tetracyclic 12, 13 – epoxytrichothecene skeleton plays a major role in the cytotoxicity of DON [43,44]. The EDR3 transforms the epoxide group of DON to nontoxic metabolite de-epoxy-DON (DOM-1) by epoxidase produced from *Eubacterium BBSH 797* (Table 1) [45,46]. The compositions of EDR1, EDR2, and EDR4 also contain epoxidase (Table 1) that degrades the tetracyclic 12, 13 – epoxytrichothecene skeleton of DON. Zearalenone is a powerful xenoestrogen by the activation of estrogen receptors [47]. Ji et al. [46] suggested the lactonase could catalyze the hydrolysis of ZEN, followed by spontaneous decarboxylation. The compositions of EDR1, EDR2 contain lactonase (Table 1) that degrades the lactone ring of ZEN. The metabolism of ZEN by *Trichosporon mycotoxinivorans* was investigated by Vekiru et al. [48]. Therefore, *T. mycotoxinivorans* of EDR3 (Table 1) is responsible for the observed ZEN degrading ability in EDR3. According to the patent of EDR5 claims, *Bacillus subtilis ANSB471* and *B. subtilis ANSB01G* of EDR5 have DON and ZEN degraded ability, but the patent does not elaborate on the degradation mechanism.

The DON and ZEN removabilities of EDRs differed significantly with different pH values for AGJ and AIJ. This result can be explained by the fact that EDRs mainly work on enzymatic hydrolysis, and the enzyme activity is determined by the pH value of AGI and AIJ [49]. Schatzmayr et al. [45] indicated that the de-epoxidation activity of *E. BBSH 797* and *Trichosporon mycotoxinivorans* could be verified under conditions of the intestinal environment within 48 h where the active culture developed DON and ZEN-detoxification activity in all tested intestinal segments. The best result was obtained in the anterior part of the small intestine, where 100% of added DON were transformed, resulting in 100% diepoxy-deoxynivalenol. In the respective control samples, biotransformation activity was not detected. From these results, it could be assumed that the intestinal environment regarding the intestinal microflora, as well as the physiological conditions including pH, redox potential, and oxygen content, generally enables biotransformation of DON and ZEN by *E. BBSH 797* and *T. mycotoxinivorans* [45]. The patent of EDR4 (US 10,221,403 B1, USA) indicated zearalenone hydrolase has the best ZEN removability in a neutral buffer (pH 6.0 to 8.3), and the patents of EDR5 (CN103243047A and CN102181376B, China) suggested that *Bacillus subtilis ANSB471* and *ANSB01G* have the best DON and ZEN removabilities in a neutral buffer as well. These patents explain why EDR3–EDR5 have higher DON and ZEN removability in the intestine than in the stomach. Up to date, there is no information available for pH values of enzymes used in EDR1, EDR2, and EDR4. In our research results, EDR1 and EDR2 were more suitable in the monogastric animal stomach of pH 2.5 to 4.5; EDR4 was more suitable in the monogastric animal intestine of pH 6.5. The removabilities of DON and ZEN for EDRs showed more significant increases within 1 h. After 1 h, the removability of EDRs did not rise linearly over time and differed significantly according to the pH value of the AGJ and AIJ. Our results suggest that EDR1 and EDR2 operating in AGJ at a pH of 2.5 and 4.5 in the stomach of monogastric animals are the most suitable mycotoxin removers. On the other hand, EDR3 and EDR4 were more suitable with AIJ at a pH of 6.5 of AIJ for the small intestines of monogastric animals.

Although DON and ZEN concentrations were significantly degraded in the in vitro simulated gastrointestinal systems of pig and poultry by EDRs, we did not analyze the metabolites degraded from the toxin in this study. Our results indicate that EDRs are capable of degrading DON and ZEN under the experimental conditions used here, but it is unclear if the metabolite residues of DON and ZEN are still toxic as DON and ZEN themselves. Future studies should be conducted to further evaluate the effects of nutrition adsorption of EDRs and adsorbents in commercial feeds. Furthermore, the residual AGJ and AIJ could be used to culture intestinal epithelial cells of pig and poultry to evaluate the toxicities of metabolites to assess whether or not mycotoxin removers achieve genuine detoxification. It is necessary to validate if cell toxicities of in vitro digestion metabolites of mycotoxins still exit. Further, the effectiveness of EDRs and adsorbents in reducing DON and ZEN toxic effects without affecting the regular utilization of essential nutrients, such as water-soluble vitamins and minerals needs to be addressed.

## 4. Conclusions

In the present study, we demonstrated that EDRs are robust and effective mycotoxin removers based on pig and poultry gastrointestinal simulations. Under the same contamination level of mycotoxins, DON and ZEN removabilities of EDRs are more effective than that of adsorbents in the simulated gastrointestinal tracts. Furthermore, the high recovery rates and low CVs in AGJ and AIJ conditions verify the accuracy and stability of our procedures. In terms of preventing mycotoxicosis, these simulations are not only useful for evaluating the mycotoxin removability of various mycotoxin removers, in addition to EDRs and adsorbents, but also for recapitulating the condition that most closely mimic natural environments of the gastrointestinal tracts in pig and poultry species.

## 5. Materials and Methods

### 5.1. Reagents

DON and ZEN standards, with the purity higher than 99%, were purchased from Fermentek (Jerusalem, Israel). Acetonitrile and methanol were HPLC grade and purchased from Merck (Darmstadt, Germany). The analytical pure grade agents, such as porcine pepsin, porcine trypsin, porcine bile salt, 36.5% hydrochloric acid (HCl), monopotassium phosphate (KH_2_PO_4_), sodium chloride (NaCl), sodium hydroxide (NaOH), and polyethylene glycol (PEG, molecular weight 8000) were purchased from Sigma–Aldrich (Saint Louis, MO, USA). The mycotoxin removers included five EDRs and two adsorbents. We ensured that the products used in the study were the ones most relevant to farmers and feed mills and could be purchased commercially. The suppliers included international companies, such as Patent (Mišićevo, Serbia), Amlan (Chicago, IL, USA), Henan Yi-Wan Zhong-Yuan (Henan, China), Biomin (Inzersdorf–Getzersdorf, Austria), Liferainbow (Yilan, Taiwan), Dr. Bata (Budapest, Hungary), for the main contents of mycotoxin removers as shown in Table 1. The commercial sow feed and breeding hen feed used in the in vitro procedure were kindly provided by Farmwealth (Tainan, Taiwan), and the raw material composition and nutrient value are shown in Table 3.

### 5.2. Solution Preparation

#### 5.2.1. Standards Preparation of DON and ZEN

The DON and ZEN standards were prepared as described by Cahill et al. [50] and Arranz et al. [51], respectively. One milligram of the DON standard powder was dissolved in 10 mL of methanol, which was then made into 100 mg/L and kept at −20 °C as a storage solution for further use. The storage solution was diluted to 0.5, 1, and 5 mg DON/L using acetonitrile–water (10:90, *v*/*v*) for DON standard curves. One milligram of the ZEN standard powder was dissolved in 10 mL of acetonitrile, which was then made into 100 mg/L ZEN standard and kept at −20 °C for further use. The storage solution was diluted to 0.025, 0.25, 0.5, and 1 mg ZEN/L using acetonitrile–water–methanol (46:46:8, *v*/*v*) for ZEN standard curves. The R^2^ values obtained from all the DON and ZEN standard curves were > 0.9990.

#### 5.2.2. Preparation of AGJ and AIJ

The AGJ and AIJ were prepared as described by Wang et al. [26]. Two grams of NaCl and 3.2 g of pepsin were initially dissolved in sufficient water. Then 5 mL of 36.5% HCl was added, and the obtained mixture was diluted to 1000 mL with more water to obtain AGJ. The pH of the AGJ was adjusted to 2.5 with 0.1 M NaOH. The solution was kept at 4 °C for further use. Six-point eight grams of KH_2_PO_4_ was dissolved in 500 mL water, and the pH of the resulting solution was adjusted to 6.8 with 0.1 M NaOH. Ten grams of trypsin was dissolved in water, mixed with the KH_2_PO_4_ solution, and then 3 g of porcine bile salt was added and diluted with water to 1000 mL to obtain AIJ. The pH of the AIJ was adjusted to 6.5 with 0.1 M NaOH and 36.5% HCl. The solution was kept at 4 °C for further use.

### 5.3. In Vitro Procedure

#### 5.3.1. Simulation of Pig Gastrointestinal Tracts

**Figure 5:**
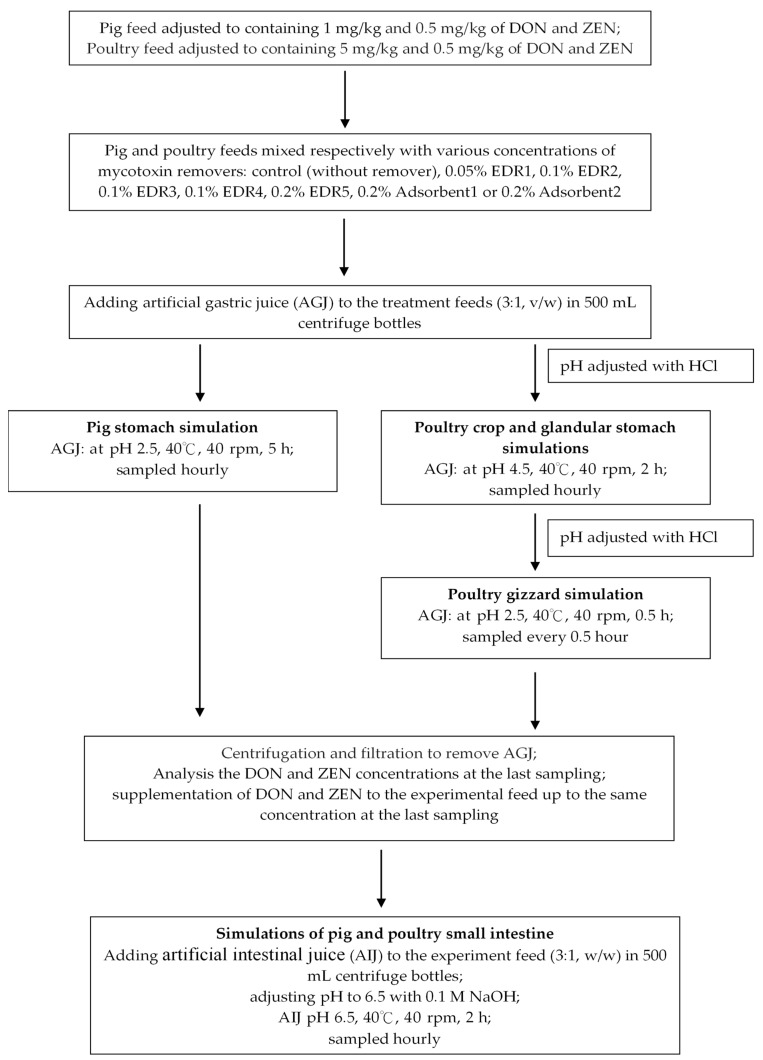
The procedures of pig and poultry in vitro gastrointestinal simulations. DON: Deoxynivalenol; EDR: Enzyme degradation reagents; ZEN: Zearalenone.

The conditions pH and time for pig gastrointestinal tract were modified according to Avantaggiato et al. [28] and Wang et al. [26]. The procedure is described in Figure 5. Commercial pig feed was analyzed to determine the initial concentration of DON and ZEN by HPLC. Then, we added the DON and ZEN standards into the feed to adjust the DON and ZEN concentration to 1 mg/kg and 0.5 mg/kg, respectively. Two hundred grams of adjusted DON and ZEN concentration of pig feed was pre-mixed with the mycotoxin remover as control. The various mycotoxin remover concentrations were as follows: 0.05% EDR1, 0.1% EDR2, 0.1% EDR3, 0.1% EDR4, 0.2% EDR5, 0.2% Adsorbent1, and 0.2% Adsorbent2. Sixty grams of treated feed was weighed and placed into a 500 mL centrifuge tube, and AGJ was added at a ratio of 1:3 (*w*/*v*). The pH of the resulting solution was adjusted to 2.5 ± 0.2 with 36.5% HCl, and the solution was incubated at a constant temperature in a shaking incubator at 40.0 ± 1 °C and 40.0 ± 5 rpm for 5 h. In this stage, the stomach digestion conditions of pigs were simulated. Twenty milliliters of AGJ per treatment per hour was immediately and respectively added to the DON and ZEN extraction solution to inhibit the reactions of digestive enzymes in the EDRs and adsorbents. After 5 h of incubation, the treatment solution was centrifuged and filtered to remove AGJ and keep the residual treatment feed at −20 °C for inhibiting the reactions of digestive enzymes, EDRs, and adsorbents. In the fifth hour, the AGJ sample of each treatment was immediately extracted and analyzed to determine the concentration of DON and ZEN by HPLC. Then the DON and ZEN standards were added to the feed to adjust the DON and ZEN concentration to AGJ at 5 h. The AIJ was added at a ratio of 1:3 (*w*/*v*). The pH of the resulting solution was adjusted to 6.5 ± 0.2 with 0.1 M NaOH, and the solution was incubated in a constant temperature in a shaking incubator at 40.0 ± 1 °C and 40.0 ± 5 rpm for 2 h; this stage simulated the small intestine digestion of pigs. Twenty milliliters of AIJ per treatment per hour was immediately added to the DON and ZEN extraction solution. In addition, the validation of accuracy and precision were determined based on rates of recovery of DON and ZEN, which were added (at three replicates) to the pig feed at four concentrations at 0.5, 1, 2, and 5 mg/kg and 0.025, 0.25, 0.5, and 1 mg/kg for ZEN. Twenty milliliters of AGJ for 5 h and 20 mL of AIJ for 2 h were collected for DON and ZEN analyses. The recovery rate was calculated by comparing the peak areas of mycotoxin standards and mycotoxins in the feed released in the simulated digestive juice.

#### 5.3.2. Simulation of Poultry Gastrointestinal Tracts

The pH simulation conditions for the gastrointestinal tract of poultry were modified, according to Zyla et al. [25]. The procedure is described in Figure 5. We extracted and analyzed the concentration of DON and ZEN by HPLC for commercial poultry feed. Then, we added the DON and ZEN standards to the feed to adjust the DON and ZEN concentration to 5 mg/kg and 0.5 mg/kg, respectively. Two hundred grams of adjusted DON and ZEN concentration of poultry feed was premixed with mycotoxin remover as control. The various concentrations of mycotoxin removers were as following: 0.05% EDR1, 0.1% EDR2, 0.1% EDR3, 0.1% EDR4, 0.2% EDR5, 0.2% Adsorbent1, and 0.2% Adsorbent2. Sixty grams of treated feed was added to a 500 mL centrifuge tube, and AGJ was added at a ratio of 1:3 (*w*/*v*). The pH of the resulting solution was adjusted to 4.5 ± 0.2 with 36.5% HCl, and the solution was incubated at a constant temperature in a shaking incubator at 40.0 ± 1 °C and 40.0 ± 5 rpm for 2 h. Then, 20 mL of AGJ per treatment per hour was directly added to the DON and ZEN extraction solution, respectively. After 2 h of incubation, the treatment solution was adjusted to 2.5 ± 0.2 with HCl, and the solution was incubated at a constant temperature in a shaking incubator at 40.0 ± 1 °C at 40.0 ± 5 rpm for 0.5 h. Then, 20 mL of AGJ per treatment per hour was directly added to the DON and ZEN extraction solution. After 2.5 h of incubation, the treatment solution was centrifuged and filtrated to remove AGJ and keep the residual treatment feed at −20 °C. After 2.5 h, an AGJ sample of each treatment was immediately extracted and analyzed to determine the concentrations of DON and ZEN by HPLC. After this process, the DON and ZEN standards were added to the feed to adjust the DON and ZEN concentration in AGJ at 2.5 h. The AIJ was added at a ratio of 1:3 (*w*/*v*). The pH of the resulting solution was adjusted to 6.7 ± 0.2 with 0.1 M NaOH, and the solution was incubated at a constant temperature in a shaking incubator at 40.0 ± 1 °C at 40.0 ± 5 rpm for 2 h. Then, 20 mL of AIJ per treatment per hour was directly added to the DON and ZEN extraction solutions. Exactly the same validation and design were applied in poultry feed at four concentrations: 0.5, 1, 2, and 5 mg/kg for DON and 0.025, 0.25, 5, and 1 mg/kg for ZEN. Twenty milliliter of AGJ at a pH of 4.5 for 2 h, 20 mL of AGJ at pH 2.5 for 0.5 h, and 20 mL of AIJ at pH 6.5 for 2 h were collected for DON and ZEN analyses. The recovery rate was calculated by comparing the peak areas of mycotoxin standards and mycotoxins in the feed released in the simulated digestive juice.

#### 5.3.3. Extraction and Chromatographic Conditions of DON

The DON analysis was carried out and modified according to methods in Cahill et al. and Janes et al. [50,52]. The crude extraction procedures for the feed sample and artificial digestive juice differed slightly, but the purification and HPLC conditions were the same. For crude extraction of feed sample, 20 g ground feed, 8 g PEG, and 80 mL water were mixed in a 250 mL quartz cup. The mixed samples were blended at 40,000 rpm for 5 min by a homogenizer (PT-MR 3000, Brinkmann, Germany). Extracts were filtered through both a filter paper (Whatman#1, Whatman, England) and a glass fiber filter (Vicam#31955, Vicam, Milford, MA, USA). For crude extraction of artificial digestive juice, 10 mL AGJ or AIJ with 1 g PEG and 10 mL water were mixed in 50 mL centrifuge tube then shaken for 2 min by vortex (Vortex Genie 2, Scientific Industries, Bohemia, NY, USA). Extracts were then filtered through a glass microfiber filter.

For the DON purification of samples, 2 mL of accurately measured filtrate was applied to a DONTest^TM^ affinity column (Vicam, Milford, MA, USA), and DON bound specifically to the antibody. Subsequently, the column was washed with 5 mL water, and DON was eluted by passing 2 mL methanol through the column and collecting this eluate in a glass cuvette. The eluate was dried using a nitrogen concentrator (Pierce React-Therm III^TM^, Thermo Fisher Scientific, Waltham, MA, USA), re-dissolved in 2 mL acetonitrile–water (10:90, *v*/*v*) and shaken for 1 min by vortex. The resulting solution was then filtered with a 0.45 μm syringe filter (Micron Separations, Westborough, MA, USA). The filtrate was kept at 4 °C for HPLC analysis. For the HPLC conditions of our DON analysis, 50 μL standards or sample filtrate were injected using an auto-sampler (L-2200, Hitachi, Tokyo, Japan) into an HPLC pump (L-2130, Hitachi, Japan) with the UV detector set to 220 nm. The acetonitrile–water (10:90, *v*/*v*) mobile phase was filtered through a 0.45 μm filter membrane (Micron Separations, Westborough, MA, USA), degassed, and used at a flow rate of 1 mL/min. The analytical column used was Mightysil^TM^ C18 column (4.6 × 150 mm, 5μm, Kanto Chemical, Tokyo, Japan).

#### 5.3.4. Extraction and Chromatographic Conditions of ZEN

The ZEN analysis was carried out and modified according to Arranz et al. [51]. The crude extraction procedures for the feed sample and artificial digestive juice were slightly different, but the purification and HPLC conditions were the same. For crude extraction of feed samples, 20 g ground feed sample was added to 5 g NaCl and 50 mL acetonitrile–water (90:10, *v*/*v*), and they were mixed in a 250 mL quartz cup and made homogeneous after 2 min. Extracts were then filtered through filter paper and a glass fiber filter; then, a 10 mL filtered extract was transferred into another clean vessel, and the extract was diluted with 40 mL of deionized water. The diluted extract was then filtered through a glass fiber filter into a clean vessel. For the crude extraction of artificial digestive juice, 10 mL AGJ or AIJ with 1 g NaCl and 10 mL acetonitrile–water (90:10, *v*/*v*) were mixed in a 50 mL centrifuge tube and then shaken for 2 min by vortex. Extracts were then filtered through a glass microfiber filter.

For the purification of ZEN samples, 10 mL of filtrate was accurately measured and then applied to a ZearalaTest™ affinity column (Vicam, Milford, MA, USA), and ZEN bound specifically to the antibody. Subsequently, the column was washed with 10 mL water, and the ZEN was eluted by passing it through 1.5 mL of methanol through the column and collecting the resulting eluate in a glass cuvette. Then, 1.5 mL water was added for dilution, vortexed for 1 min, and then filtered using a 0.45 μm syringe filter. The filtrate was kept at 4 °C for HPLC analysis.

For the HPLC conditions of the ZEN analysis, 100 μL standards or sample filtrate were injected into an HPLC system with a fluorescence detector (L-2480, Hitachi, Tokyo, Japan) set to Ex 274 nm and Em 440 nm. The acetonitrile–water–methanol (46:46:8, *v*/*v*/*v*) mobile phase was filtered through a 0.45 μm filter membrane, degassed, and used at a flow rate of 1 mL/min.

### 5.4. Statistical Analysis

The DON and ZEN removabilities of each mycotoxin removal agent were estimated as a percentage of the total mycotoxins removed from the AIG and AJI solutions, represented by Equation (1).

(1) $$Y=\frac{\left(\mathrm{CC}-\mathrm{CT}\right)}{\mathrm{CC}}\times 100\%$$

where Y is DON or ZEN removability; CC is the concentration of free toxins in the gastrointestinal tract system of the control group; CT is the concentration of free toxins in the gastrointestinal tract system of the treatment group. This calculation method can offset the matrix effect of AGJ and AIJ. The DON and ZEN concentration of the control group is shown in Table 4. The statistical analysis of DON and ZEN removability was performed as a completely randomized design using the PROC GLM general factorial ANOVA procedure, using SAS version 9.4 (SAS Institute Inc., Cary, NC, USA). Statistically significant effects were further analyzed, and means were compared using Tukey’s HD test. Statistical significance was determined at *p* ≤ 0.05.

## Figures and Tables

**Table 1:** Composition, product information, and correct patent of mycotoxin removers.

Mycotoxin Remover	Dosage (%)	Brand/Company	Composition (Patent)
EDR1	0.05	Detoxa Plus^®^ New/Dr. Bata	Yeast cell wall, lactonase (NCAIM (P) Y001470), de-epoxidase (NCAIM (P) Y001469), carboxypeptidase (NCAIM (P) Y001468) and carboxylesterase (NCAIM (P) Y001467)
EDR2	0.10	Detoxa Plus^®^/Dr. Bata	Yeast cell wall, lactonase (NCAIM (P) Y001470), de-epoxidase (NCAIM (P) Y001469), carboxypeptidase (NCAIM (P) Y001468) and carboxylesterase (NCAIM (P) Y001467)
EDR3	0.10	Micofix Plus^®^MTV/Biomin	Bentonite (EC 1060/2013), *Eubacterium BBSH797* (EC 1016/2013), FUMzyme^®^ (EC 1115/2014), *Trichosporon mycotoxinivorans* and plant-origination components
EDR4	0.10	Toxi-free^®^/Liferainbow	HSCAS, zearalenone hydrolase (US 10,221,403 B1), epoxidase and peptidase
EDR5	0.20	MLJ^®^/Henan Yi-Wan Zhong-Yuan	Yeast cell wall, the mixed enzymes were produced by 3 *Bacillus subtilis* which contained *ANSB471* (CN 103,243,047 B), *ANSB01G* (CN 102,181,376 B) and *ANSB060* (CN 101,705,203 B)
Adsorbent1	0.20	Calibrin-Z^®^/Amlan	Activated calcium montmorillonite (US 20160030475 A1)
Adsorbent2	0.20	Minazel Plus^®^/Patent	Clinoptilolite (EP 1,363,854 B1)

EDR: Enzyme degradation reagents; HSCAS: Hydrated sodium calcium aluminosilicate.

**Table 2:** Recovery rates of mycotoxins from artificial gastric juice (AGJ) and artificial intestinal juice (AIJ) of pig and poultry gastrointestinal simulations.

Mycotoxin	Concentration mg/kg	Pig				Poultry					
		AGJ (pH 2.5, 5 h)		AIJ (pH 6.5, 2 h)		AGJ (pH 4.5, 2 h)		AGJ (pH 2.5, 0.5h)		AIJ (pH 6.5, 2 h)	
		Recovery%	CV%	Recovery%	CV%	Recovery%	CV%	Recovery%	CV%	Recovery%	CV%
DON	0.500	90.0	4.23	94.4	4.23	91.9	5.05	89.8	5.13	88.8	5.17
	1.000	90.8	3.30	94.5	2.23	92.9	4.81	91.3	5.15	90.5	5.32
	2.000	94.0	2.13	89.8	2.64	93.1	4.92	90.8	5.72	89.7	6.12
	5.000	95.3	2.44	86.4	4.75	93.2	2.80	90.8	2.82	89.6	2.83
ZEN	0.025	92.5	4.01	85.0	4.25	92.1	3.89	90.7	3.78	90.0	3.73
	0.250	95.2	4.54	84.7	4.28	90.0	4.81	87.3	4.86	85.9	4.89
	0.500	95.5	4.09	88.4	4.84	92.1	5.58	90.3	5.74	89.4	5.82
	1.000	95.9	2.34	90.5	4.80	93.6	5.38	92.5	6.09	92.0	6.45

Values are the means of three replicates. Limit of detection of DON and ZEN are 0.1 and 0.01 mg/kg, respectively. CV: Coefficient of variation; DON: Deoxynivalenol; ZEN: Zearalenone.

**Table 3:** The compositions of diets for in vitro simulations.

Item	Feed	
	Pig Feed	Poultry Feed
Ingredients, %		
Corn	667	570
Soybean meal, 44%	200	200
Full fat soybean	60	80
Limestone	-	99
Wheat middling	-	25
Fish meal, 65% crude protein	20	-
Monocalcium phosphate	-	18
Diphosphate	25	-
Calcium	20	-
Salt	4	2
Sodium bicarbonate		3
Choline chloride		1
^1^Vitamin premix	2	1
^2^Mineral premix	2	1
Calculated values		
ME, kcal/kg	3250	2707
Crude protein, %	17.50	17.14
Crude fate, %	4.021	3.878
Calcium, %	1.568	4.053
Total phosphorus, %	0.875	0.722
Available phosphorus, %	0.548	0.392
Copper, ppm	56.96	33.54
Zinc, ppm	225.4	166.2
Iron, ppm	359.4	224.2

^1^Vitamin premix: Each kg contained retinol 3 g, cholecalciferol 0.06 g, D-α-tocopherol 18.0 g, thiamine 1 g, riboflavin 4.5 g, pyridoxine 3.2 g, cobalamin 0.01 g, biotin 0.2 g, menadione 1.5 g, D-calcium pantothenate 10 g, folic acid 0.5 g, and nicotinic acid 25 g. ^2^Mineral premix: Each kg contained copper 15.0 g, ferrum 80 g, zinc 50 g, manganese 80 g, cobalt 0.25 g, and iodine 0.85 g.

**Table 4:** The deoxynivalenol (DON) and zearalenone (ZEN) concentration of control group for in vitro simulations.

Simulation Model	Simulation Time (h)	DON (mg/kg)	ZEN (mg/kg)
Pig	0	1.00 ± 0.008	0.50 ± 0.021
	1	1.00 ± 0.109	0.48 ± 0.080
	2	0.99 ± 0.050	0.48 ± 0.058
	3	0.96 ± 0.127	0.47 ± 0.027
	4	0.94 ± 0.128	0.43 ± 0.069
	5	0.91 ± 0.117	0.42 ± 0.002
	6	0.90 ± 0.103	0.41 ± 0.009
	7	0.85 ± 0.146	0.40 ± 0.043
Poultry	0	5.00 ± 0.154	0.50 ± 0.149
	1	4.83 ± 0.607	0.50 ± 0.006
	2	4.64 ± 0.420	0.50 ± 0.055
	2.5	4.38 ± 0.282	0.49 ± 0.004
	3.5	4.17 ± 0.282	0.48 ± 0.058
	4.5	3.93 ± 0.165	0.47 ± 0.089

DON: Deoxynivalenol; EDR: Enzyme degradation reagents; ZEN: Zearalenone.
